# Worse sleep health predicts less frequent breakfast consumption among adolescents in a micro-longitudinal analysis

**DOI:** 10.1186/s12966-022-01265-5

**Published:** 2022-06-17

**Authors:** Gina Marie Mathew, David A. Reichenberger, Lindsay Master, Orfeu M. Buxton, Lauren Hale, Anne-Marie Chang

**Affiliations:** 1 Program in Public Health, Department of Family, Population, and Preventive Medicine, Renaissance School of Medicine, Stony Brook University, Health Sciences Center, 101 Nicolls Road, Level 3, Room 071, Stony Brook, NY 11794-8338 USA; 2 Department of Biobehavioral Health, College of Health and Human Development, Pennsylvania State University, University Park, PA USA

**Keywords:** Sleep duration, Sleep timing, Sleep maintenance efficiency, Subjective sleep quality, Sleep variability, Social jetlag, Actigraphy, Diary, Breakfast, Adolescence

## Abstract

**Background:**

Poor self-reported sleep health has been linked to not consuming breakfast in adolescents, but it is unknown whether poor sleep measured objectively predicts next-day breakfast consumption within adolescents. We investigated within- and between-person associations of objectively measured sleep dimensions and subjective sleep quality with adolescent breakfast consumption.

**Methods:**

Data were collected from a micro-longitudinal substudy of the Year 15 wave of the Fragile Families and Child Wellbeing Study (*n* = 590). Adolescents wore an actigraphy device and completed daily diaries for ~ 1 week (*M* ± *SD* = 5.6 ± 1.4 nights per adolescent, range: 3–9), where they rated their sleep quality and reported whether they had eaten breakfast that day, with no specific definition of breakfast provided (*M* ± *SD* = 5.5 ± 1.4 days per adolescent, range: 3–9). Separate mixed models assessed whether actigraphy-measured sleep duration (linear and quadratic, sleep duration x sleep duration), timing, maintenance efficiency, and subjective quality predicted odds of breakfast consumption both within and between adolescents. Variability of sleep duration and timing (standard deviation per person), sleep regularity index (SRI), and social jetlag were tested as additional between-person predictors. Analyses with predictors other than sleep duration were adjusted for sleep duration.

**Results:**

Following nights when adolescents had shorter or longer sleep duration (*p* = .005; curvilinear association), later sleep onset, or later sleep midpoint (both *p* = .025) than their own usual, they had lower odds of consuming breakfast the next day (within-person associations). Adolescents who on average had later sleep onset (*p* = .013) or midpoint (*p* = .013) or who reported lower sleep quality (*p* = .011) had lower average odds of consuming breakfast (between-person associations). Adolescents with greater variability of sleep duration (*p* = .005), midpoint (*p* = .004), or offset (*p* < .001) had lower average odds of consuming breakfast (between-person associations). Sleep maintenance efficiency (within or between adolescents), SRI, and social jetlag were not associated with breakfast consumption (all *p* > .10).

**Conclusions:**

Multiple dimensions of sleep health are associated with breakfast consumption, both within and between adolescents. Poor sleep and dietary behaviors in adolescence may negatively impact future metabolic health.

**Supplementary Information:**

The online version contains supplementary material available at 10.1186/s12966-022-01265-5.

## Background

Breakfast consumption has positive associations with health and wellbeing and is associated with lower risk of obesity [1] and better cardiometabolic health [2, 3]. Breakfast consumption is also linked with greater self-reported alertness [4], better academic performance [5], and better psychological health in adolescents [6]. Interventions that increase breakfast consumption in adolescents increase performance during cognitive tasks [7] and during exercise [8] and result in less weight gain compared to not consuming breakfast [9]. Breakfast consumption may improve alertness and performance in adolescents due to the positive effects of glucose on attention [10], psychological health by providing nutrients that are beneficial to mental health such as magnesium [11], and cardiometabolic health by decreasing total and LDL cholesterol [2]. The definition of breakfast varies among individuals; some may define breakfast as a meal before or at the start of daily activities, while others may define breakfast as a meal consumed before a certain time (e.g., before 10:00) [12]. Identifying risk factors of reduced breakfast consumption, particularly modifiable behaviors, may be beneficial for physical health and functioning, especially given that approximately 32% of adolescents do not consume breakfast daily [13], and nearly 60% do not consume breakfast 3 or more times per week [14].

Self-reported dimensions of sleep, such as duration and timing, have been linked to breakfast consumption in adolescents [15–23]. Adolescents tend to have a later chronotype, or preference for timing of sleep and other behaviors, than children or adults [15]. Later self-reported chronotype is cross-sectionally associated with reduced odds of consuming breakfast [16] and not consuming breakfast 67% or more of the time [17] in adolescents. Over 70% of adolescents report obtaining fewer than the recommended minimum of 8 h per night [24], and adolescents who report receiving fewer than 7 h [18], 8 h [19–21], or 8.5 h [22] are less likely to consume breakfast regularly. Longer self-reported sleep is also linearly associated with more frequent breakfast consumption [23]. Interestingly, some research indicates the opposite association. One study found that adolescents who reported obtaining fewer than 8 h of sleep one night were more likely to consume breakfast the next day [25], while another demonstrated that adolescents who reported getting fewer than 8 h of sleep nightly were more likely to have breakfast 6 days out of the week [26]. These equivocal findings indicate the need for more research with objective measures of sleep.

Sleep health is a multidimensional construct that also includes objective quality (e.g., sleep efficiency), subjective quality, and variability [27], all of which may also predict breakfast consumption. For example, worse subjective sleep quality [16] and greater self-reported social jetlag [28] (a misalignment in sleep timing across the week) have been associated with reduced breakfast consumption in adolescents. However, there is minimal research examining the associations of objective sleep quality and variability in sleep duration and timing with breakfast consumption in adolescents. Also, there is a lack of research examining this topic with objective sleep measures, and self-reported sleep can deviate considerably from sleep assessed through objective measures such as actigraphy [29].

Most studies that examine the association between sleep and breakfast consumption in adolescents employ a cross-sectional design [16–23, 26, 28], which precludes examination of within-person effects. Whereas between-person effects describe the characteristics of the individual adolescent (e.g., examining whether adolescents who sleep longer on average tend to consume breakfast more often than other adolescents) [30], within-person effects can establish temporal precedence (e.g., examining whether adolescents are more likely to consume breakfast the morning after nights when they sleep longer than usual) [31]. Between-person effects do not necessarily translate to within-person effects in direction or magnitude [30], warranting the examination of both types of effects.

The present study examined whether objectively measured sleep dimensions and subjective sleep quality predicted breakfast consumption both within (Aim 1) and between (Aim 2) adolescents. We additionally examined whether sleep variability was associated with greater breakfast consumption between adolescents (Aim 3). We hypothesized that both long and short sleep duration, later sleep timing, lower sleep maintenance efficiency, and lower subjective sleep quality would be associated with lower breakfast consumption within and between adolescents, and greater sleep variability would be associated with lower breakfast consumption between adolescents.

## Methods

### Participants

Data for the current analyses come from the Fragile Families and Child Wellbeing Study (FFCWS; www.fragilefamilies.princeton.edu), a longitudinal birth cohort oversampled for nonmarital births, which resulted in a greater proportion of racial/ethnic minority mothers and those of lower socioeconomic status and education level compared to the national population. More details regarding the sample and design may be found elsewhere [32]. This study was conducted according to the guidelines laid down in the Declaration of Helsinki of 1975 (revised 1983), and all procedures involving human participants were approved by the Princeton University and Stony Brook University (CORIHS B) (FWA #00,000,125) Institutional Review Boards. Written (for in-home interviews) or recorded verbal (for phone interviews) informed consent was obtained from primary caregivers, and assent was obtained from adolescents.

The original FFCWS birth cohort consists of 4,898 children born from 1998–2000 in 20 large U.S. cities [33]. Families were recruited from local hospitals at the time of the child’s birth. The study staff maintained records about the participants and their families for follow up at subsequent waves, when participants were approximately ages 1, 3, 5, 9, and 15 years of age. Families were eligible for inclusion in the Year 15 follow-up wave if the child was alive, not legally adopted, and participated in the year 9 wave. Data in the current analyses were collected from February 2014 to March 2016. During the Year 15 wave of the FFCWS (wave 6), 3,444 adolescents and their primary caregivers completed separate surveys querying household and demographic characteristics, administered either over the phone or in person at the participant’s place of residence. The research firm Westat® used random sampling methods to select a subsample (*N* = 1,090), who were asked to participate in a micro-longitudinal FFCWS substudy [34]. Adolescents who agreed to participate (*N* = 1,049) were asked to wear a wrist-worn accelerometer and answer a daily diary for seven consecutive days in the evening. Out of 1,049 assenting adolescents, *n* = 419 were excluded due to not providing at least 3 valid nights of actigraphy recordings (see the "Wrist actigraphy" section; current sample *M* ± *SD* = 5.6 ± 1.4 nights per adolescent; range 3–9; interquartile range, IQR 5–7) and next-day daily diary reports (current sample *M* ± *SD* = 5.5 ± 1.4 days per adolescent; range 3–9; IQR 4–7), and *n* = 40 were excluded due to missing covariate values (see below for covariate descriptions), leaving a total sample of *N* = 590 adolescents (56.2% of the subsample). An additional *n* = 218 adolescents were excluded from social jetlag analyses due to not providing data from at least one school night and one free night, resulting in *n* = 372 included adolescents. Supplemental Figure S1 depicts the participant flow chart, and a “Strengthening the Reporting of Observational Studies in Epidemiology – Nutritional Epidemiology” (STROBE‐nut) checklist is included as Supplemental Table S1.

Separate logistic regression analyses were conducted examining whether sex, race/ethnicity, and income predicted exclusion from the present analyses due to data missingness (included *n* = 590; excluded *n* = 459). Male sex (OR = 1.35, *p* = .017), Black/African American race/ethnicity (vs. White/Caucasian; OR = 1.83, *p* = .001), and lower household income (in thousands of dollars; OR = .997, *p* = .003) predicted higher odds of data missingness. All analyses adjusted for these demographic characteristics.

## Materials and measures

### Wrist actigraphy

Sleep measures were collected with a wrist-worn accelerometer with off-wrist detection (Actiwatch Spectrum; Philips-Respironics, Murrysville, PA) and study participants were asked to wear the watch on their non-dominant wrist for one week. Data from the Spectrum device were downloaded with Philips Actiware software (Version 6.0.4, Philips Respironics, 2017). Accelerometer devices measure movements, from which patterns of sleep and wake may be estimated [35]. At least two independent scorers (blinded to each other) determined cut-point (i.e., start and end time that defines a 24-h day), validity of days, and sleep intervals with a duration of 30 min or more using a validated procedure [36]. A program was used to compare differences between the scorers in the determination of the number of valid days, cut-point, number of sleep intervals, and any > 15-min differences in duration or wake after sleep onset (WASO) for each recording. The scorers adjudicated all differences, and a third scorer was consulted for difficult recordings. The scorers determined sleep intervals using a decrease in activity levels and the aid of light levels for sleep onset and sleep offset [37], and a nighttime sleep interval was split into two intervals (main sleep and nap) if there was an awakening ≥ 1 h during this interval. No sleep intervals were set if the duration was less than 30 min. A sleep actigraphy day was determined invalid and no sleep interval was set if there were ≥ 4 total hours of off-wrist time, with the exception of the first and last day (device should have been worn at least 2 h on the first day). Other invalidation criteria were constant false activity due to battery failure, data unable to be retrieved or recovered, or an off-wrist period of ≥ 60 min within 10 min of the scored beginning or end of the main sleep period for that day. Nights were excluded from current analyses if the adolescent had an all-nighter (i.e., no sleep interval was set within that 24-h day; *n* = 2 nights).

#### Nightly sleep measures

*Sleep onset* and *sleep offset* were the start and end of the main nighttime sleep interval (i.e., when an adolescent fell asleep at night and woke up the next morning, respectively) calculated in hours from midnight, respectively.

*Sleep duration* was calculated as the number of hours between sleep onset and sleep offset of the main nighttime sleep interval.

*Sleep midpoint* (also midnight-centered) was calculated as midway between sleep and sleep offset.

*Sleep maintenance efficiency* represents the percent of sleep duration that the individual spent asleep and was calculated as *1 – (WASO in hours / sleep duration in hours)* and multiplied by 100 to produce a percentage [38–41].

#### Actigraphic sleep measures calculated per adolescent

We additionally calculated measures of sleep variability per adolescent. *Sleep duration variability*, *sleep onset variability*, *sleep midpoint variability*, and *sleep offset variability* were calculated as the standard deviation (*SD*) of each measure per adolescent.

*Sleep regularity index* (SRI) was calculated based on the formula from Phillips et al. [42] and ranges from 0 (low regularity) to 100 (high regularity). The score represents the percentage probability that an individual is in the same state (sleeping or awake) at any two time points 24 h apart, averaged across the interval of measurement.

*Social jetlag*, a misalignment of sleep midpoint between school and free days, was calculated in hours through the following formula: | sleep midpoint on free nights − sleep midpoint on school nights | [43]. Only adolescents with at least one school night and one free night were included in the social jetlag measure; therefore, some adolescents did not have social jetlag values (*N* = 372 of the 590 total adolescents had social jetlag values).

### Daily diary

Participants were asked to complete a diary each evening after 7:00 PM (19:00) and before going to sleep for all 7 days of the week. The questions in the diary asked about last night’s sleep through the current day, and adolescents were not permitted to complete the diary after they went to bed that night (i.e., the next day). The diary queried information regarding last night's sleep (including subjective sleep quality) and napping that day, that day’s school attendance and start time, whether the adolescent was sick, medications used, exercising behavior, communication and conflict with friends and family, electronic device usage, work performed, mood, and dietary intake (including breakfast consumption) that day. Participants were permitted to skip items if they did not wish to answer, and skipped items were treated as missing data. Sleep and daily diary data were merged by participant identification number and date/time. A “day” was defined as spanning from sleep onset to the next sleep onset (typically about 24 h). Therefore, a previous night’s sleep and the following day’s diary answers were aligned on the same observation record. If there was not a main sleep onset at the end of a day due to an invalid sleep period (i.e., device removed), the day ended when it encountered more than 1 h off-wrist time or an apparent sleep interval scored by a validated algorithm [36], whichever occurred first.

#### Subjective sleep quality

Adolescents rated their last-night sleep quality by answering, “How would you rate your sleep quality?” with possible answers of “very good” (1), “fairly good” (2) “fairly bad” (3) or “very bad” (4). This variable was reverse coded such that “very bad” became 0, and “very good” became 3.

#### Breakfast consumption

Adolescents were asked, "Did you eat breakfast?", with “no” coded as 0 and “yes” coded as 1. In order to accommodate differences in social norms, cultural behaviors, and individual habits in meal timing, content, and portion size [44], no specific definition of "breakfast" was provided.

#### School attendance

Adolescents also reported whether they went to school (0 = no school, 1 = school) that day.

#### Mood

Adolescents reported their mood that day on a scale created by the FFCWS substudy team. Specifically, they answered how “bored,” “lonely,” and “happy” they felt during that day with possible answers of “very slightly or not at all” (1), “a little” (2), “moderately” (3), “quite a bit” (4), or “extremely” (5), which were recoded into 0 = “not at all” to 4 = “extremely.”

### Physical measurements

During the home visit, height and weight were measured by the trained interviewer. Height measurements were taken in centimeters using a stadiometer. Weight measurements were taken in pounds using a scale. The interviewer took two measurements each, and a third measurement was taken if the first two measurements differed. Height and weight were used to calculate body mass index (BMI): *weight in kg / (height in m*^*2*^*)* [45]. The BMI percentile variable (range: 0–100) was constructed using the Centers for Disease Control and Prevention’s (CDC) statistical program, which calculates the percentiles for the adolescent's sex and age based on the 2000 CDC growth charts [46].

### Year 15 surveys

Other characteristics were assessed at birth or through surveys administered once to adolescents and their primary caregivers during the Year 15 wave.

#### Demographic and household characteristics

Sex information was collected at birth. Race/ethnicity was reported on the Year 15 survey and was grouped into exclusive categories of White/Caucasian (not Hispanic or Latino), Black/African (not Hispanic or Latino), Hispanic and/or Latino (any race), or a category with other (including Asian, Central American/Caribbean, Native American/Alaska Native, and/or Native Hawaiian/Pacific Islander), mixed, and no race/ethnicity. Adolescent living arrangements (i.e., whether the adolescent lived with two biological parents or not) were also reported on the survey. Annual household income (in USD) and the primary caregiver's highest education level (did not complete high school, completed high school, completed some college, or college graduate) were reported on the primary caregiver survey.

#### Depressive symptoms

Depressive symptoms were assessed through a modified five-item version of the Center for Epidemiologic Studies Depression Scale [47]. Adolescents selected level of agreement with six statements expressing depressive symptoms: “strongly agree” (1), “somewhat agree” (2), “somewhat disagree” (3), and “strongly disagree” (4). A depressive symptoms score was calculated as the average of item scores and reverse scored, with greater composite score indicating greater depressive symptomology (i.e., 0 = strongly disagree, 3 = strongly agree).

## Statistical analyses

Analyses were conducted in SAS 9.4 software (SAS Institute, Cary, North Carolina). Most variables met standards for normality (skew <|3| and kurtosis <|10|) [48]. Variability of sleep onset, midpoint, and offset (*SD*s) and social jetlag were positively skewed (skew ≥ 3) and/or leptokurtotic (kurtosis ≥ 10) and were winsorized (i.e., values beyond the 99^th^ percentile were replaced with the 99^th^ percentile value). Of 590 adolescents, 6 sleep onset *SD* values, 6 sleep midpoint *SD* values, 7 sleep offset *SD* values, and 1 social jetlag value were winsorized. After winsorization, these variables also met criteria for normality.

### Analyses among dimensions of sleep

We used linear mixed models (PROC MIXED) to test within-person associations and Pearson correlations to test between-person associations among the dimensions of sleep (see Table S2).

### Associations of adolescent characteristics with breakfast consumption

Multilevel models (PROC GLIMMIX, with a binary distribution for the outcome) were used to test the associations of potential covariates with breakfast consumption: school attendance, mood (boredom, loneliness, and happiness), depressive symptoms, sex, race/ethnicity, BMI percentile, household income, adolescent living arrangements, and primary caregiver highest education level completed.

### Main analyses

Multilevel models (PROC GLIMMIX, with a binary distribution for the outcome) were used to test whether dimensions of nightly sleep (sleep duration, sleep onset, midpoint, and offset, and sleep maintenance efficiency, measured through actigraphy; subjective sleep quality, measured through diary) predicted the odds of breakfast consumption, both within and between adolescents. To test whether there was a curvilinear relationship between sleep duration and breakfast consumption (i.e., whether both short and long sleep predicted breakfast consumption), a quadratic association between sleep duration and odds of breakfast consumption was assessed using the predictor sleep duration^2^ (sleep duration*sleep duration). Additional between-person models separately tested whether variability of sleep duration, onset, midpoint, and offset, SRI, and social jetlag, measured through actigraphy, were associated with breakfast consumption. For the sleep measures that varied within persons (sleep duration, onset, midpoint, offset, maintenance efficiency, and subjective sleep quality), two-level models examined 3,250 total daily observations that were clustered within 590 adolescents. Of those 3,250 observations, 4 values of subjective sleep quality were not reported on the daily diary, leaving *n* = 3,246 observations for analyses with the predictor subjective sleep quality. Variances for nightly sleep measures were decomposed into within-person (level-1) and between-person (level-2) levels [31]. Within-person sleep predictor variables were centered around the person mean, such that positive values indicated that value was higher than the person’s own cross-day average. Between-person sleep predictor variables were calculated as the mean per person across all time points. For the sleep variability measures that were between-person only (*SD* of duration, onset, midpoint, and offset, SRI, and social jetlag), the sleep variability measure predicted average odds of breakfast consumption per adolescent. We specified the denominator degrees of freedom to be computed by dividing the residual degrees of freedom into between-subject and within-subject portions (DDFM = BETWITHIN) [49], used AR(1) covariance structure, and included a random intercept for participant variation. Covariates that were significantly associated with odds of breakfast consumption were included in all models: school attendance, boredom, loneliness, happiness, sex, race/ethnicity, household income, BMI percentile, and depressive symptoms score. Adolescent living arrangements (*p* = .076) and primary caregiver education level (*p* = .688) were not associated with breakfast consumption and were therefore not adjusted for in main analyses. Analyses with predictors other than sleep duration were further adjusted for sleep duration and sleep duration^2^. Alpha < .05 (two-sided) was deemed statistically significant.

## Results

### Demographic characteristics

In total, 590 adolescents provided at least 3 nights of actigraphy, next-day diary, and complete covariate data (53% female, *n* = 315; mean ± *SD* age = 15.4 ± .5 years, range 14.7–17.7). Ethnoracial composition of the sample was as follows: 41% Black/African American (*n* = 241), 25% non-Hispanic or Latino (*n* = 148), 19% White/Caucasian (*n* = 112), and 15% other, mixed, or none (*n* = 89). The mean percent of days that adolescents reported consuming breakfast was 70% (see Table 1 for other sample information, including descriptive statistics for sleep variables of interest and covariates). Not attending school that day (within-person; OR = .63, *p* < .001), male sex (OR = 1.98, *p* < .001), other, mixed, or no race (compared to White/Caucasian, non-Hispanic; OR = 1.80, *p* = .038), higher income (OR = 1.003, *p* = .016), and lower BMI percentile (OR = .99, *p* < .001) were associated with increased average odds of breakfast consumption (see Table 2).

**Table 1:** Average descriptive statistics for analytical sample (*N* = 590)

Variable	*M* or *%*	*(SD* or *n)*
Demographic and household		
Sex^a^		
Female	53%	(315)
Male	47%	﻿(275)
Race/ethnicity		
Black/African American	41%	﻿(241)
Hispanic and/or Latino	25%	﻿(148)
White/Caucasian	19%	﻿(112)
Other,^b^ mixed, or none	15%	﻿(89)
Body mass index (BMI) percentile^c^	73.88	﻿(24.96)
Annual household income	$65,794	﻿($61,776)
Adolescent living arrangements		
Does not live with two biological parents	68%	﻿(401)
Lives with two biological parents	32%	﻿(189)
Primary caregiver highest education completed		
Less than high school	14%	﻿(81)
High school	18%	﻿(105)
Some college	47%	﻿(276)
College	22%	﻿(128)
Emotional health		
Boredom^d^	1.19	﻿(.88)
Loneliness^d^	.52	﻿(.76)
Happiness^d^	2.19	﻿(.90)
Depressive symptoms score^e^	.60	﻿(.59)
School attendance		
Attended school (proportion of days)	.43	﻿(.34)
Nightly sleep measures^f^		
Sleep duration (hrs)	7.80	﻿(1.07)
Sleep onset (clock time)	0:27	﻿(1:44)
Sleep midpoint (clock time)	4:21	﻿(1:42)
Sleep offset (clock time)	8:19	﻿(1:46)
Sleep maintenance efficiency (%)	90.71	﻿(3.39)
Subjective sleep quality^g^	2.35	﻿(.50)
Sleep variability measures^h^		
Variability (*SD*) of sleep duration (hrs)	1.56	﻿(.80)
Variability (*SD*) of sleep onset (hrs)	1.29	﻿(.73)
Variability (*SD*) of sleep midpoint (hrs)	1.22	﻿(.65)
Variability (*SD*) of sleep offset (hrs)	1.57	﻿(.91)
Sleep regularity index^i^	48.49	﻿(13.24)
Social jetlag (hrs)^j^	1.80	﻿(1.15)
Dietary intake		
Consumed breakfast (proportion of days)^k^	.70	﻿(.33)

^a^Data collected at birth

^b^Other category includes Asian, Central American/Caribbean, Native American/Alaska Native, and/or Native Hawaiian/Pacific Islander

^c^Calculated based on 2000 Centers for Disease Control and Prevention (CDC) growth charts, matched for age and sex[46]

^d^Ranges from 0 (very slightly or not at all)–4 (extremely)

^e^Computed from Center for Epidemiologic Studies Depression Score [47]. Range: 0 (low)–3 (high)[47]

^f^The mean number of valid actigraphy nights per adolescent was 5.6 ± 1.4 (range: 3–9); IQR 5–7

^g^Ranges from 0 (very bad)–3 (very good)

^h^Higher value means greater variability, except the reverse for the sleep regularity index

^i^Calculated based on formula from Phillips et al. [42]; ranges from 0 (low)–100 (high)

^j^Calculated based on formula from Wittmann et al. [43]. *N* = 372 (adolescent included only if provided at least one weekday and one weekend night of actigraphy; *n* = 372)

^k^The mean number of breakfast reports per adolescent was 5.5 ± 1.4 (range: 3–9; IQR 4–7)

*M*, mean; hrs, hours; *n,* number; *SD*, standard deviation

**Table 2:** Associations of adolescent characteristics with breakfast consumption (*N* = 590)

	**Within-Person**			**Between-Person**		
**Model predictor**	**OR**	**95%CI OR**		**OR**	**95%CI OR**	
Daily measures (diary)						
Attended school (ref = no school)	.63***	[.50	.80]	.83	[.52	1.32]
Boredom^a^	.99	[.90	1.10]	.75**	﻿[.62	.89]
Loneliness^a^	.99	[.87	1.14]	.75**	﻿[.60	.92]
Happiness^a^	1.01	[.91	1.13]	1.42***	﻿[1.19	1.68]
Person-level measures (one-time survey)						
Male (ref = female)^b^	---	---	---	1.98***	﻿[1.46	2.69]
				**OR**	**95%CI OR**	
Black/African American (ref = White/Caucasian)	---	---	---	.90	﻿[.59	1.39]
Hispanic and/or Latino (ref = White/Caucasian)	---	---	---	1.23	﻿[.77	1.97]
Other,^c^ mixed, or none (ref = White/Caucasian)	---	---	---	1.80*	﻿[1.03	3.14]
Body mass index (BMI) percentile^d^				.99***	﻿[.98	1.00]^e^
Depressive symptoms score^f^				.77*	﻿[.59	1.00]^g^
Household income	---	---	---	1.00*^h^	﻿[1.00	1.01﻿]
Adolescent lives with two bio. parents (ref = no)	---	---	---	1.35	﻿[.97	1.89]
				**OR**	**95%CI OR**	
High school (ref = less than high school)	---	---	---	.87	﻿[.50	1.50]
Some college (ref = less than high school)	---	---	---	.92	﻿[.58	1.47]
College graduate (ref = less than high school)	---	---	---	1.15	﻿[.67	1.95]

*Notes.* Each row represents a separate linear mixed model. The within-person effect is represented by the deviation from the adolescent’s overall mean at each time point (daily diary measures only). The between-person effect is represented by each adolescent’s mean across all time points. The mean number of breakfast reports was 5.5 ± 1.4 (range: 3–9; IQR 4–7) per adolescent

^a^Ranges from 0 (very slightly or not at all)–4 (extremely)

^b^Data collected at birth

^c^Other category includes Asian, Central American/Caribbean, Native American/Alaska Native, and/or Native Hawaiian/Pacific Islander

^d^Calculated based on 2000 Centers for Disease Control and Prevention (CDC) growth charts, matched for age and sex[46]

^e^95%CI upper limit = .995

^f^Computed from Center for Epidemiologic Studies Depression Score [47]. Range: 0 (low)–3 (high)[47]

^g^95%CI upper limit = .998

^h^OR = 1.003; 95%CI lower limit = 1.0006

^*^p < .05, **p < .01, ***p < .001, two-tailed

bio biological,* CI* confidence interval, *OR* odds ratio, *ref* reference category

## Associations of sleep measures with breakfast consumption

### Nightly sleep measures (within-person associations; Aim 1)

**Fig. 1:**
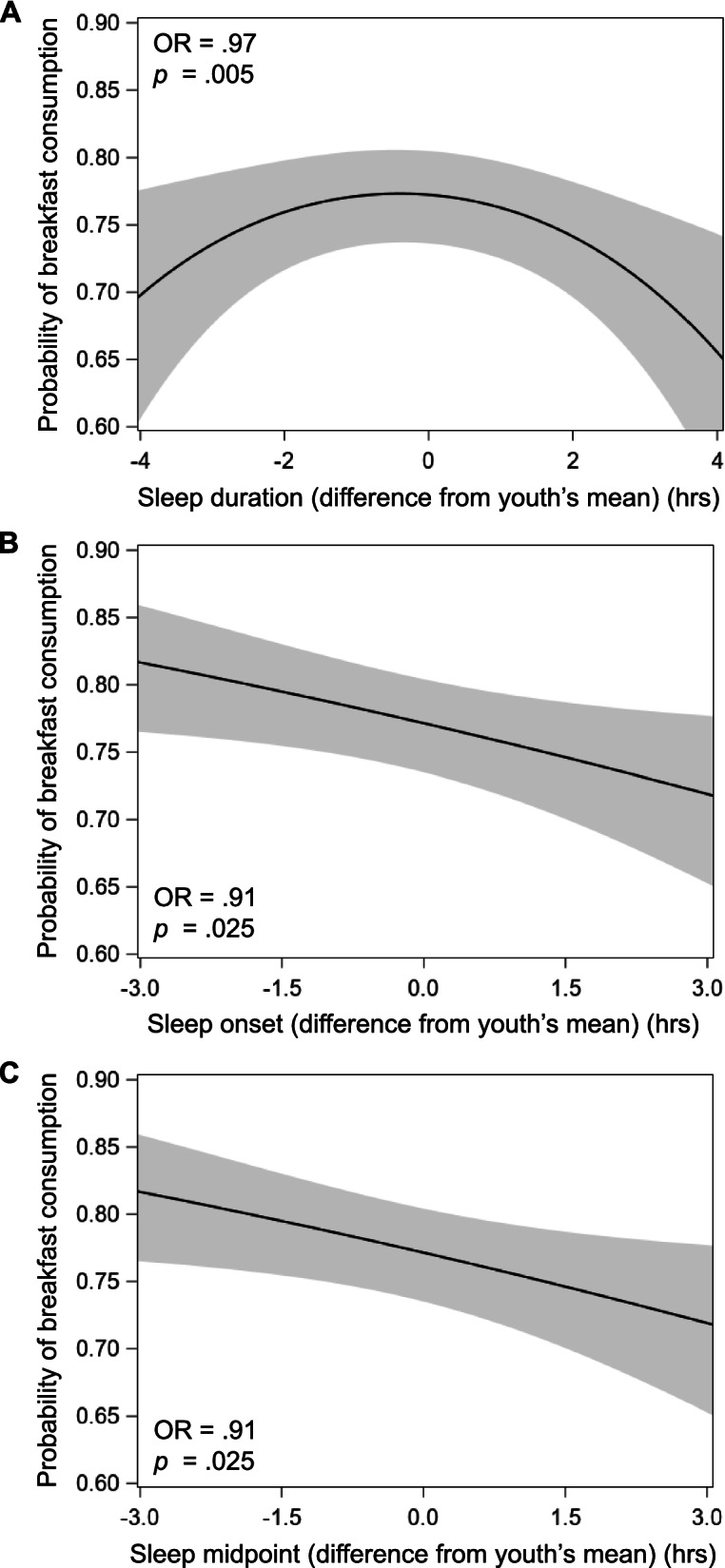
Associations of deviation from adolescent’s mean sleep duration (**A**), sleep onset (**B**), and sleep midpoint (**C**) (each in hours) and probability of next-day breakfast consumption (within-person associations) in three separate mixed models. Sleep duration model includes both linear (sleep duration) and quadratic (sleep duration^2^) effects (only the significant quadratic effect is depicted). Sleep onset and sleep midpoint models adjust for sleep duration (linear and quadratic, sleep duration^2^). The mean number of valid actigraphy nights was 5.6 ± 1.4 (range: 3–9; IQR 5–7) and the mean number of breakfast reports was 5.5 ± 1.4 (range: 3–9; IQR 4–7) per adolescent. All models adjust for demographic/household covariates: school day, boredom, loneliness, happiness, birth sex, race/ethnicity, household income, body mass index percentile, and depressive symptoms. Shaded bands depict 95% confidence interval of probability of breakfast consumption predicted from each sleep measure

There was a quadratic within-person association between sleep duration (i.e., sleep duration^2^) and next-day breakfast consumption (*p* = .005) in fully adjusted analyses, such that following nights when an adolescent received an hour shorter or longer sleep than their own usual, their odds of consuming breakfast the next day decreased by 3% (see Table 3 and Fig. 1). The linear within-person association between sleep duration and breakfast consumption was not significant (*p* = .420). There were significant within-person associations between both sleep onset and midpoint with breakfast consumption (both *p* = .025; Fig. 1). Following nights when an adolescent had a sleep onset or midpoint an hour later than their own usual, they had 9% lower odds of consuming breakfast the next day. There were no significant within-person associations of sleep offset (*p* = .080), sleep maintenance efficiency (*p* = .430), or subjective sleep quality (*p* = .762) with breakfast consumption.

**Table 3:** Associations of dimensions of sleep with breakfast consumption (*N* = 590)

	**Within-Person**			**Between-Person**		
**Model predictor**	**OR**	**95%CI OR**		**OR**	**95%CI OR**	
Nightly sleep measures						
Sleep duration (linear, hrs)	.98	[.92	1.04]	.55	[.14	2.22]
Sleep duration*sleep duration (quadratic, hrs)	.97**	[.95	.99]	1.03	[.95	1.13]
Sleep onset (hrs)	.91*	[.84	.99]	.88*	[.80	.97]
Sleep midpoint (hrs)	.91*	[.84	.99]	.88*	[.80	.97]
Sleep offset (hrs)	.94^†^	[.87	1.01]	.91^†^	[.82	1.01]
Sleep maintenance efficiency (%)	.99	[.96	1.02]	1.03	[.98	1.08]
Subjective sleep quality^a^	1.03	[.85	1.25]	1.58*	[1.11	2.24]
Sleep variability measures^b^						
Sleep duration (*SD*, hrs)	---	---	---	.75**	[.61	.92]
Sleep onset (*SD*, hrs)	---	---	---	.82^†^	[.65	1.04]
Sleep midpoint (*SD*, hrs)	---	---	---	.68**	[.53	.88]
Sleep offset (*SD*, hrs)	---	---	---	.74***	[.62	.88]
SRI^c^	---	---	---	1.01	[1.00	1.02]
Social jetlag (hrs)^d^	---	---	---	.96	[.80	1.15]

*Notes.* Each row represents a separate multilevel model that adjusts for demographic/household covariates: school day (within-person; nightly measures only), boredom, loneliness, happiness, birth sex, race/ethnicity, household income, body mass index percentile, and depressive symptoms (all between-person). Models with predictors of interest other than sleep duration further adjust for sleep duration (linear and quadratic, sleep duration^2^). The within-person effect for nightly sleep measures is represented by the deviation from the adolescent’s overall mean at each time point. The between-person effect for nightly sleep measures is represented by each adolescent’s mean across all time points. The between-person effect for sleep variability measures is represented by *SD*, SRI, or social jetlag value per adolescent. Sleep timing measures (onset, midpoint, and offset) were centered around midnight (0:00). The mean number of valid actigraphy nights was 5.6 ± 1.4 (range: 3–9; IQR 5–7) and the mean number of breakfast reports was 5.5 ± 1.4 (range: 3–9; IQR 4–7) per adolescent

^a^Ranges from 0 (very bad)–3 (very good)

^b^Higher value means greater variability, except the reverse for the SRI

^c^Calculated based on formula from Phillips et al. [42]; ranges from 0 (low)–100 (high)

^d^Calculated based on formula from Wittmann et al. [43]. *N* = 372 (adolescent included only if provided one weekday and one weekend night of actigraphy; *n* = 372)

^†^*p* < .10, *p < .05, **p < .01, ***p < .001, two-tailed

*CI* confidence interval, *hrs* hours, *OR* odds ratio, *SRI* sleep regularity index

### Nightly sleep measures (between-person associations; Aim 2)

**Fig. 2:**
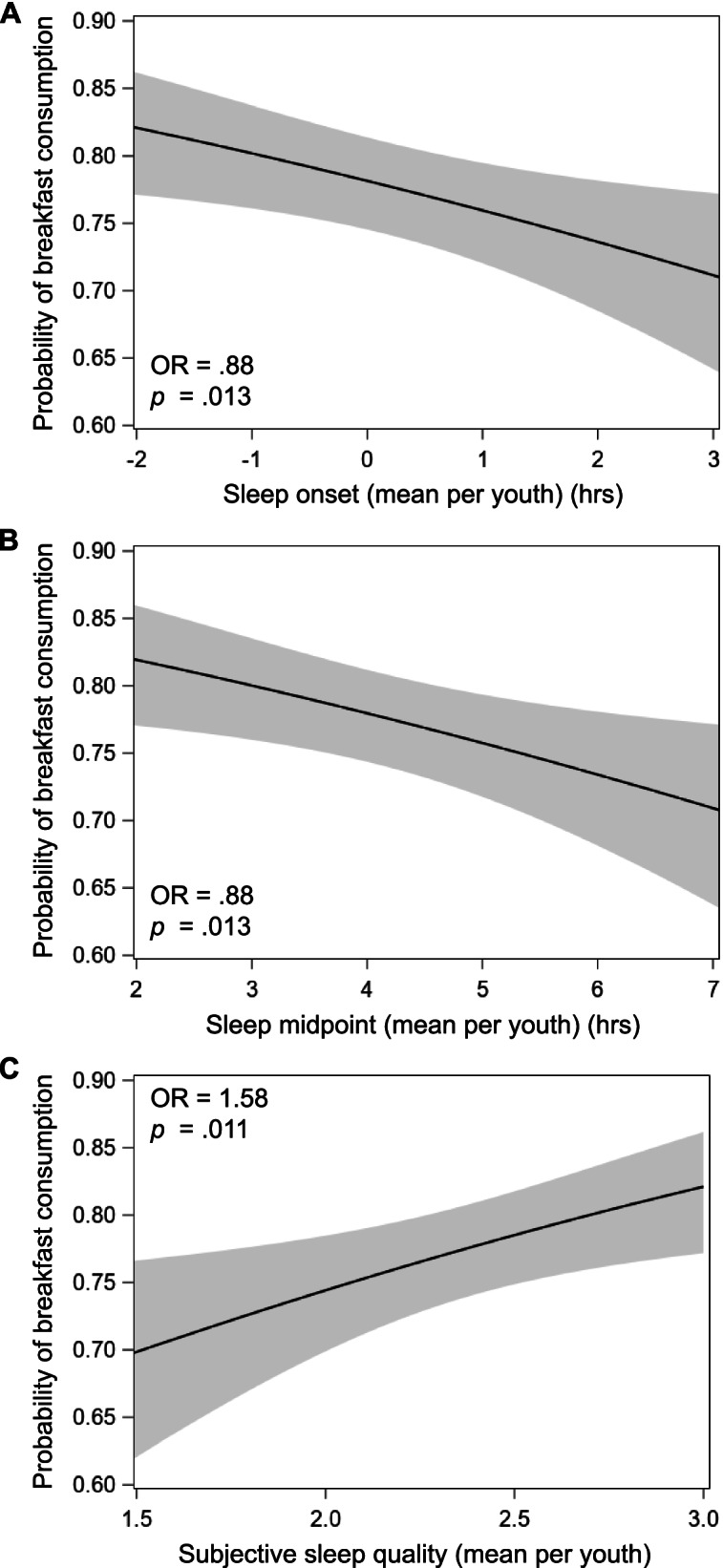
Associations of average sleep onset in hours (**A**), sleep midpoint in hours (**B**), and subjective sleep quality (**C**) per adolescent with average probability of breakfast consumption (between-person associations) in three separate linear mixed models. Each effect is represented by the adolescent’s mean across all time points. Sleep onset and midpoint were centered around midnight (0:00). Subjective sleep quality ranges from 0 (very bad) to 3 (very good). The mean number of valid actigraphy nights was 5.6 ± 1.4 (range: 3–9; IQR 5–7) and the mean number of breakfast reports was 5.5 ± 1.4 (range: 3–9; IQR 4–7) per adolescent. All models adjust for sleep duration (linear and quadratic, sleep duration^2^) and demographic/household covariates: school day, boredom, loneliness, happiness, birth sex, race/ethnicity, household income, body mass index percentile, and depressive symptoms. Shaded bands depict 95% confidence interval of probability of breakfast consumption predicted from each sleep measure

There were significant between-person associations between both sleep onset and midpoint with breakfast consumption (both *p* = .013; see Table 3 and Fig. 2), such that adolescents who had a one-hour later sleep onset or midpoint versus others had 12% lower odds of consuming breakfast on average. There was a significant between-person association between subjective sleep quality and breakfast consumption (*p* = .011), such that adolescents who reported one-unit greater sleep quality versus others had 58% greater odds of consuming breakfast on average (Fig. 2). There were no significant between-person associations of sleep duration (linear: *p* = .404; quadratic: *p* = .450), sleep offset (*p* = .064), or sleep maintenance efficiency (*p* = .200) with breakfast consumption.

### Sleep variability measures (between-person associations; Aim 3)

**Fig. 3:**
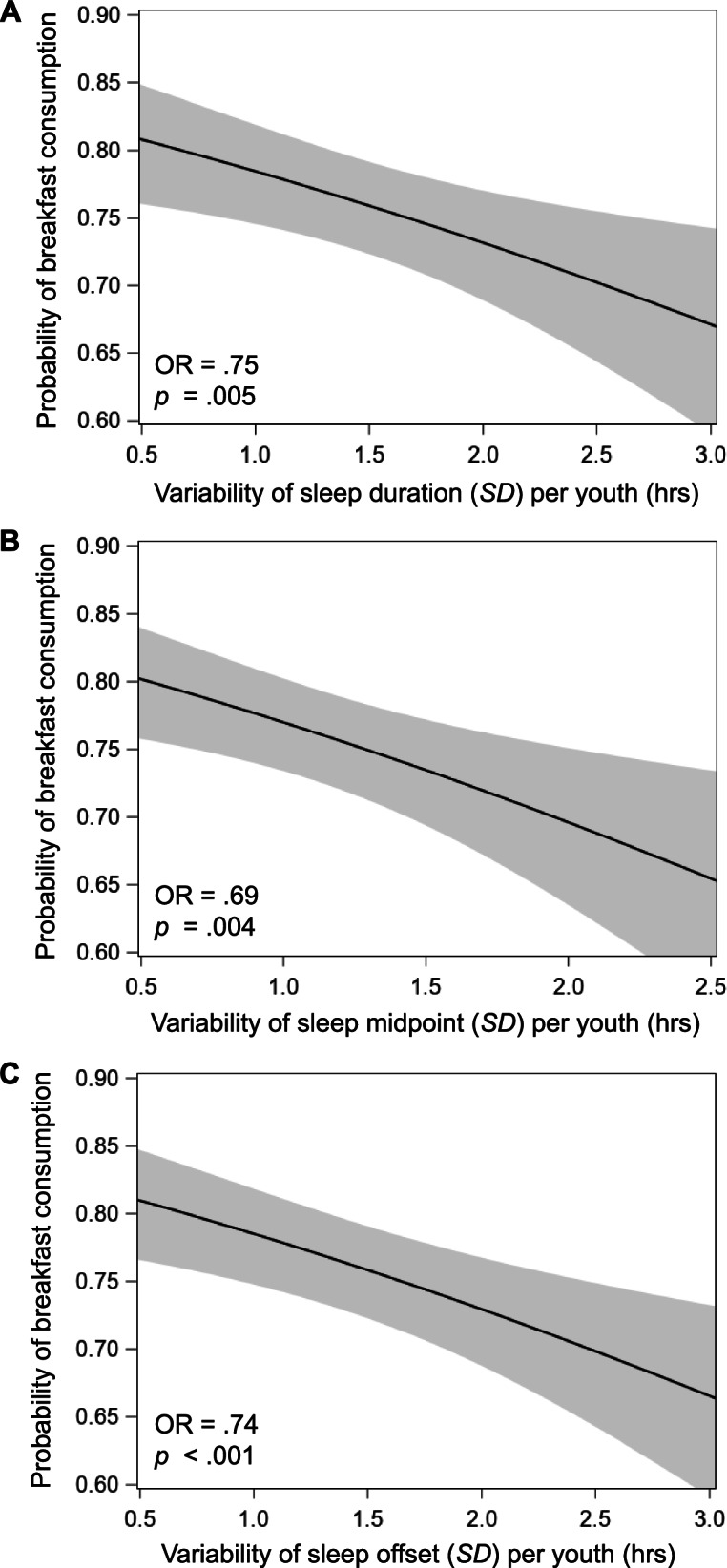
Associations of variability in sleep duration (**A**), sleep midpoint (**B**), and sleep offset (**C**) (calculated as standard deviation, *SD*, of hours) per adolescent with average probability of breakfast consumption (between-person associations) in three separate linear mixed models. Each predictor is represented by each adolescent’s *SD* across all time points. The mean number of valid actigraphy nights was 5.6 ± 1.4 (range: 3–9; IQR 5–7) and the mean number of breakfast reports was 5.5 ± 1.4 (range: 3–9; IQR 4–7) per adolescent. All models adjust for sleep duration (linear and quadratic, sleep duration^2^) and demographic/household covariates: school day, boredom, loneliness, happiness, birth sex, race/ethnicity, household income, body mass index percentile, and depressive symptoms. Shaded bands depict 95% confidence interval of probability of breakfast consumption predicted from each sleep measure

There were significant associations between variability of sleep duration (*p* = .005), sleep midpoint (*p* = .004), and sleep offset (*p* < .001) with breakfast consumption, such that for every one *SD*-hour increase in variability, the odds of an adolescent consuming breakfast overall decreased by 25%, 32%, and 26%, respectively (see Table 3 and Fig. 3). There were no significant associations of sleep onset variability (*p* = .098), SRI (*p* = .126), or social jetlag (*p* = .648) with breakfast consumption.

## Discussion

The present study examined whether multiple dimensions of sleep health were associated with breakfast consumption in adolescents. We demonstrated that adolescents were less likely to consume breakfast after nights when they had shorter or longer sleep duration, later sleep onset, or later sleep midpoint than usual (within-person associations). Adolescents with later sleep onset or midpoint, lower subjective sleep quality, or more variable sleep duration, midpoint, or offset were less likely to consume breakfast, on average (between-person associations). Objective sleep quality (sleep maintenance efficiency), SRI, and social jetlag were not associated with breakfast consumption. The findings demonstrate that multiple dimensions of sleep predict breakfast consumption both within and between adolescents, offering potential targets for future interventions to improve sleep, increase breakfast consumption, and improve cardiometabolic health [2, 3].

The findings that objectively measured short sleep duration and later sleep timing were associated with reduced breakfast consumption in this large adolescent sample are consistent with previous self-report literature [16–23]. With every one hour shorter or longer sleep duration, or later sleep onset, than their own usual, adolescents were less likely to consume breakfast the next morning by 3% and 9%, respectively. While these results are statistically significant, it is not certain whether they translate to clinically relevant findings. Future research may examine whether minor increases in breakfast consumption provide meaningful impacts to physical and psychological health. Importantly, the present study is among the first to use objective measures of sleep to demonstrate temporal precedence in adolescents, such that shorter, longer, and later sleep preceded reduced breakfast consumption. Of note, only the quadratic (not the linear) association between sleep duration and breakfast consumption within adolescents was significant. The reasons why adolescents may be less likely to consume breakfast when they obtain shorter or longer sleep or have later sleep timing than their own average may be similar. Adolescents who obtain shorter sleep may have early obligations that preclude the time needed to prepare and/or eat food in the morning. In one large sample of inner-city high school students, 64% cited not having enough time in the morning as a reason for not consuming breakfast that day [50]. Indeed, adolescents were less likely to consume breakfast on school mornings in the current study, when they presumably had more demands on their time. Similarly, longer sleep duration and later sleep timing may come at the expense of time demands needed for breakfast, whether or not adolescents have school that day. Future experimental research may extend sleep duration in adolescents with short sleep and examine any impacts on breakfast consumption. Additionally, in the current study, later sleep onset was associated with shorter sleep duration within adolescents (see Table S2); on nights when adolescents fell asleep later than usual, they also had shorter sleep than usual. This suggests a potential overlap of the effects of these sleep dimensions on breakfast consumption. As we adjusted for sleep duration, the observed effects of later sleep timing on breakfast consumption in the current study were unique from those of sleep duration.

We found that variability of sleep duration and sleep offset across several days was associated with lower odds of breakfast consumption in adolescents, potentially due to the importance of routines in both regular sleep and breakfast consumption. Specifically, adolescents with a one *SD*-hour higher variability in sleep duration and offset had 25% and 26% lower average odds of consuming breakfast, respectively. Research indicates that other routine health behaviors, such as toothbrushing [51] and physical activity [52], are higher in adolescents who consume breakfast. Adolescents who lack regular sleep routines across the week may be the same adolescents who lack meal regularity; indeed, adolescents who on average had shorter sleep duration and later sleep onset also had more variability in sleep duration and sleep onset in the current study (see Table S2). Household rules may contribute to regular routines for both sleep and meal consumption, potentially resulting in longer sleep duration, earlier sleep timing, and less sleep variability across the week. For example, stricter parental rules regarding bedtimes predict more hours of weekday sleep [53], and having a household routine of eating evening meals with one's family predicts more regular breakfast consumption in adolescents [54]. Establishing routines may play a critical role for adolescents to maintain optimal sleep and physical health. A future intervention may seek to stabilize sleep schedules across the week and examine whether breakfast consumption increases in adolescents.

Subjective sleep quality was associated with breakfast consumption between but not within adolescents, suggesting that person-level characteristics may be associated with both breakfast consumption and subjective sleep quality. Adolescents with a one-unit higher subjective sleep quality had 58% greater average odds of consuming breakfast compared to others. The association persisted beyond adjustment for depressive symptoms and feelings of boredom, loneliness, and happiness, which were all associated with breakfast consumption between adolescents (all were negative associations except for happiness). Nonetheless, it is possible that some unmeasured aspect of emotional or physical wellbeing, such as lower stress [6], is associated with both better subjective sleep quality and more breakfast consumption. Alternatively, subjective sleep quality may increase breakfast consumption over the long term, resulting in lack of an observed within-person association over the course of this micro-longitudinal study. Future longitudinal research may examine whether subjective sleep quality predicts breakfast consumption over a longer time course. Interestingly, objective sleep quality was not associated with breakfast consumption between adolescents, suggesting that subjective and objective measures of sleep quality capture different dimensions of sleep health. Furthermore, in the current study, subjective sleep quality was not associated with either objective sleep duration or onset between adolescents, and analyses also adjusted for sleep duration, indicating the observed association between subjective sleep quality and breakfast consumption was unique from that of duration and onset.

Unlike *SD* of sleep duration and offset, SRI was not associated with breakfast consumption. In contrast to the *SD* measures, SRI is not specific to duration or sleep timing. Rather, SRI represents the general consistency of sleep–wake cycles across days, epoch-by-epoch, and takes into account aspects of sleep such as napping and WASO [42] (a measure of sleep continuity). From a practical standpoint, maintaining consistent sleep duration and timing across the week may be more feasible than increasing SRI and, as demonstrated by the present findings, may also be more relevant to increasing the routine healthy behavior of breakfast consumption.

In contrast to previous research in adolescents [28], social jetlag was not associated with breakfast consumption in the current study, perhaps due to smaller sample size and/or the use of objective sleep measures in the present study. A previous study that established a negative association between self-reported social jetlag and one-time measured breakfast consumption in adolescents included the current's study's participants but also an additional 2,500 individuals [28]. It is possible that the present study did not have sufficient power to detect associations between social jetlag and breakfast consumption. Alternatively, the use of self-reported sleep timing and a single question querying typical breakfast during the school week in the previous study, yet objectively measured sleep timing and daily querying of breakfast consumption in the current study, may be responsible for this difference. A related explanation is that social jetlag as measured by sleep timing on one school night versus one free night (the minimum requirement for inclusion in the current analyses) may not be representative of the adolescent’s typical social jetlag levels. Objective measurement of typical levels of social jetlag may require multiple weeks of measurement to obtain a value closer to the adolescent’s average.

The findings of the current study inform public health recommendations for pediatricians, parents, and school administrators about the potential roles of sleep duration, timing, subjective quality, and variability on healthy eating behaviors. Pediatricians may recommend that parents set regular bedtimes for their adolescent children to promote early sleep timing and to stabilize sleep schedules, which would reduce sleep variability and may increase breakfast consumption. School administrators should consider later school start times, which would allow for sufficient sleep duration and may reduce sleep variability across the week due to consistent sleep timing and reduction of social jetlag [43]. Future research may also examine whether the effects of several dimensions of sleep extend to other aspects of diet, such as consumption of obesogenic foods high in sugar and fat.

The current study has some limitations and notable strengths. Breakfast consumption was measured through daily self-report, which may not represent the adolescent’s routine eating behaviors. We also did not measure the timing or content (e.g., calories, macronutrient composition) of breakfast, which are important factors to consider in breakfast’s impact on cardiometabolic health [12, 13, 55]. Also, we cannot establish causal relationships within this observational study. A strength of the study is the large, diverse sample of adolescents across multiple areas of the United States. Future research should examine whether the present results extend to other populations, such as adolescents in other countries. Other strengths include the objective measurement of multiple dimensions of sleep with actigraphy and the examination of within-person effects, allowing for establishment of temporal precedence, which provide novel contributions to the literature.

## Conclusions

The present study demonstrated that multiple dimensions of sleep, including shorter sleep duration, later sleep timing, poorer subjective sleep quality, and sleep variability, are associated with reduced odds of breakfast consumption in a large national sample of adolescents. Improving sleep health through shifting sleep timing earlier, maintaining consistent sleep schedules, and increasing perceptions of sleep quality may be a future goal to increase breakfast consumption and improve health in adolescents.

## Supplementary Information

**Additional file 1**. Supplementary Material.

## Data Availability

Survey data from the Fragile Families and Child Wellbeing study (https://fragilefamilies.princeton.edu/documentation) are publicly available from Princeton University’s Office of Population Research (OPR) data archive: https://opr.princeton.edu/archive/restricted/Default.aspx. The sleep actigraphy and daily diary data sets generated and analyzed during the current study are not publicly available yet but will be available through an application process at the above link.

## Abbreviations

BMI: Body mass index
CDC: Centers for Disease Control and Prevention
CES-D: Center for Epidemiologic Studies Depression Scale
CI: Confidence interval
FFCWS: Fragile Families and Child Wellbeing Study
IQR: Interquartile range
LDL: Low-density lipoprotein
OR: Odds ratio
*SD*: Standard deviation
SRI: Sleep regularity index
STROBE-nut: Strengthening the Reporting of Observational Studies in Epidemiology – Nutritional Epidemiology
WASO: Wake after sleep onset
