# Supplementary material for: Worse sleep health predicts less frequent breakfast consumption among adolescents in a micro-longitudinal analysis
Source: Int J Behav Nutr Phys Act. 2022 Jun 17;19:70. doi: 10.1186/s12966-022-01265-5 (PMC9205101; doi:10.1186/s12966-022-01265-5)
Supplement: Supplementary file 1 — Additional file 1. Supplementary Material. [file 12966_2022_1265_MOESM1_ESM.pdf]

SUPPLEMENTARY MATERIAL

Title: Worse sleep health predicts less frequent breakfast consumption among adolescents in a micro-longitudinal analysis

Authors: Gina Marie Mathew, PhD,[1] David A. Reichenberger, MS,[2] Lindsay Master, MAS,[2] Orfeu M. Buxton, PhD,[2] Lauren Hale, PhD,[1] & Anne-Marie Chang, PhD[2]

Author affiliations: [1]Program in Public Health, Department of Family, Population, and Preventive Medicine, Renaissance School of Medicine, Stony Brook University, Stony Brook, NY; [2]Department of Biobehavioral Health, College of Health and Human Development, Pennsylvania State University, University Park, PA

Corresponding author: Gina Marie Mathew**,** Program in Public Health, 101 Nicolls Road, Health Sciences Center, Level 3, Room 071, Stony Brook, NY 11794-8338, (631) 216-8219, gina.mathew@stonybrookmedicine.edu

Sample Information

Data for the current analyses come from the Fragile Families and Child Wellbeing Study (FFCWS; www.fragilefamilies.princeton.edu), a longitudinal birth cohort oversampled for nonmarital births, which resulted in a greater proportion of racial/ethnic minority mothers and those of lower socioeconomic status and education level compared to the national population. More details regarding the sample and design may be found elsewhere [1].

The original FFCWS birth cohort consists of 4,898 children born from 1998-2000 in 20 large U.S. cities [2]. Families were recruited from local hospitals at the time of the child's birth. The study staff maintained records about the participants and their families for follow up at subsequent waves, when participants were approximately ages 1, 3, 5, 9, and 15 years of age. Families were eligible for inclusion in the Year 15 follow-up wave if the child was alive, not legally adopted, and participated in the year 9 wave. Data in the current analyses were collected from February 2014 to March 2016. During the Year 15 wave of the FFCWS (wave 6), 3,444 adolescents and their primary caregivers completed separate surveys querying household and demographic characteristics, administered either over the phone or in person at the participant's place of residence. The research firm Westat® used random sampling methods to select a subsample (N = 1,090), who were asked to participate in a micro-longitudinal FFCWS substudy [3]. Adolescents who agreed to participate (N = 1,049) were asked to wear a wrist-worn accelerometer and answer a daily diary for seven consecutive days in the evening. Out of 1,049 assenting adolescents, n = 419 were excluded due to not providing at least 3 valid nights of actigraphy recordings (see the "Wrist actigraphy" section; current sample $M \pm SD$ = 5.6 ± 1.4 nights per adolescent; range 3-9; interquartile range, IQR 5-7) and next-day daily diary reports (current sample $M \pm SD$ = 5.5 ± 1.4 days per adolescent; range 3-9; IQR 4-7), and n = 40 were excluded due to missing covariate values (see below for covariate descriptions), leaving a total sample of N = 590 adolescents (56.2% of the subsample). An additional n = 218 adolescents

were excluded from social jetlag analyses due to not providing data from at least one school night and one free night, resulting in *n* = 372 included adolescents. Supplemental Figure S1 depicts the participant flow chart, and a "Strengthening the Reporting of Observational Studies in Epidemiology – Nutritional Epidemiology" (STROBE-nut) checklist is included as Supplemental Table S1.

Separate logistic regression analyses were conducted examining whether sex, race/ethnicity, and income predicted exclusion from the present analyses due to data missingness (included *n* = 590; excluded *n* = 459). Male sex (OR = 1.35, *p* = .017), Black/African American race/ethnicity (vs. White/Caucasian; OR = 1.83, *p* = .001), and lower household income (in thousands of dollars; OR = .997, *p* = .003) predicted higher odds of data missingness. All analyses adjusted for these demographic characteristics.

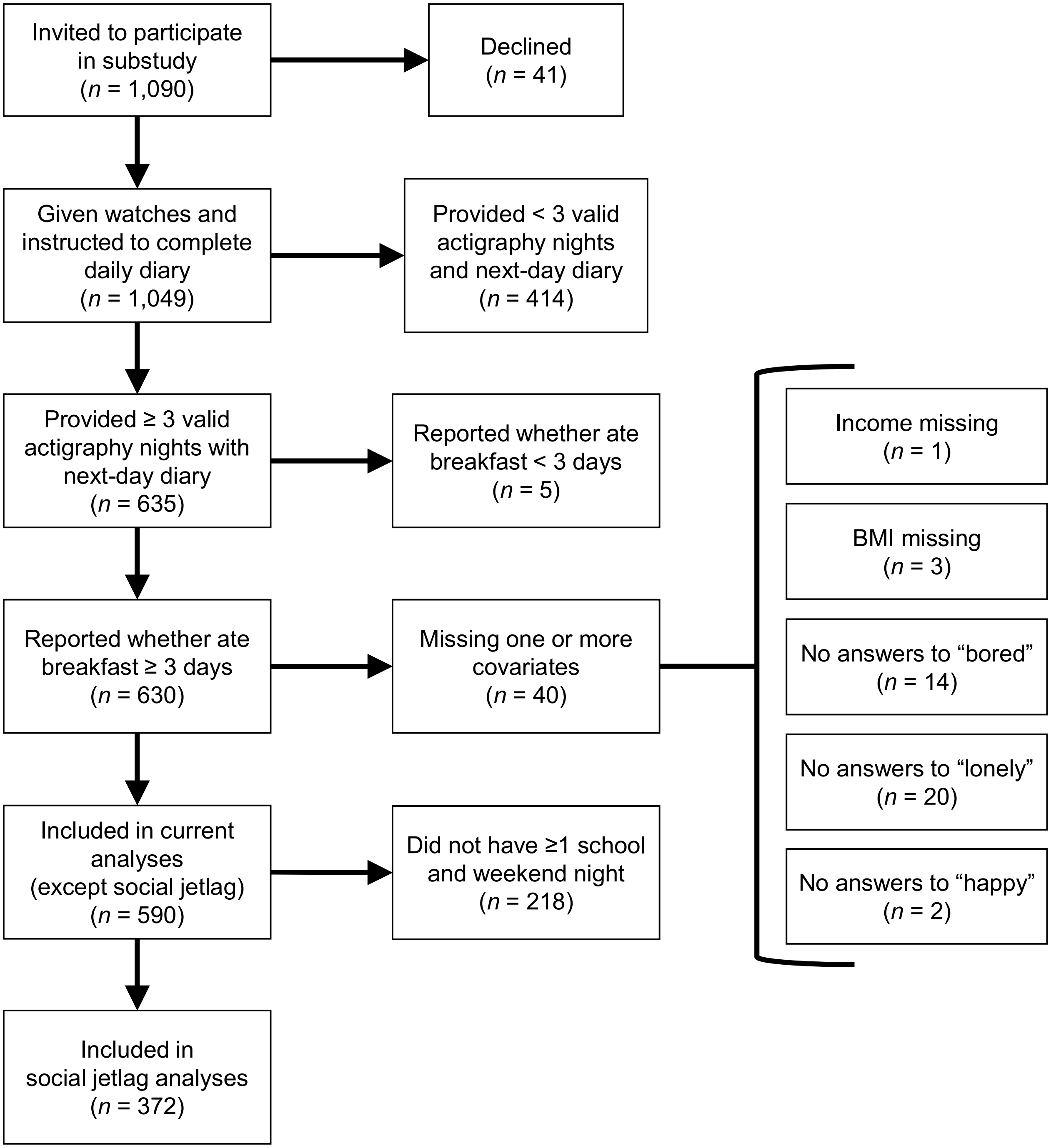

Figure S1. Participant flow chart for sample included in current analyses (*n* = 590 except for social jetlag, *n* = 372).

Table S1. STROBE-nut: An extension of the STROBE statement for nutritional epidemiology

Item	Item nr	STROBE recommendations	Extension for Nutritional Epidemiology studies (STROBE-nut)	Reported on page #
**Title and abstract**	1	(a) Indicate the study's design with a commonly used term in the title or the abstract.<br>(b) Provide in the abstract an informative and balanced summary of what was done and what was found.	**nut-1** State the dietary/nutritional assessment method(s) used in the title, abstract, or keywords.	**1**
**Introduction**				
Background rationale	2	Explain the scientific background and rationale for the investigation being reported.		**2**
Objectives	3	State specific objectives, including any pre-specified hypotheses.		**2**
**Methods**				
Study design	4	Present key elements of study design early in the paper.		**1, 3**
Settings	5	Describe the setting, locations, and relevant dates, including periods of recruitment, exposure, follow-up, and data collection.	**nut-5** Describe any characteristics of the study settings that might affect the dietary intake or nutritional status of the participants, if applicable.	**3**
Participants	6	a) Cohort study—Give the eligibility criteria, and the sources and methods of selection of participants. Describe methods of follow-up.<br>Case-control study—Give the eligibility criteria, and the sources and methods of case ascertainment and control selection. Give the rationale for the choice of cases and controls.<br>Cross-sectional study—Give the eligibility criteria, and the sources and methods of selection of participants.<br>(b) Cohort study—For matched studies, give matching criteria and number of exposed and unexposed.	**nut-6** Report particular dietary, physiological or nutritional characteristics that were considered when selecting the target population.	**3**

Item	Item nr	STROBE recommendations	Extension for Nutritional Epidemiology studies (STROBE-nut)	Reported on page #
		Case-control study—For matched studies, give matching criteria and the number of controls per case.		
Variables	7	Clearly define all outcomes, exposures, predictors, potential confounders, and effect modifiers. Give diagnostic criteria, if applicable.	**nut-7.1** Clearly define foods, food groups, nutrients, or other food components.<br>**nut-7.2** When using dietary patterns or indices, describe the methods to obtain them and their nutritional properties.	**3-5**
Data sources - measurements	8	For each variable of interest, give sources of data and details of methods of assessment (measurement).Describe comparability of assessment methods if there is more than one group.	**nut-8.1** Describe the dietary assessment method(s), e.g., portion size estimation, number of days and items recorded, how it was developed and administered, and how quality was assured. Report if and how supplement intake was assessed.<br>**nut-8.2** Describe and justify food composition data used. Explain the procedure to match food composition with consumption data. Describe the use of conversion factors, if applicable.<br>**nut-8.3** Describe the nutrient requirements, recommendations, or dietary guidelines and the evaluation approach used to compare intake with the dietary reference values, if applicable.<br>**nut-8.4** When using nutritional biomarkers, additionally use the STROBE Extension for Molecular Epidemiology (STROBE-ME). Report the type of biomarkers used and their usefulness as dietary exposure markers.<br>**nut-8.5** Describe the assessment of nondietary data (e.g., nutritional status and influencing factors) and timing of the assessment of these variables in relation to dietary assessment.<br>**nut-8.6** Report on the validity of the dietary or nutritional assessment methods and any internal or external validation used in the study, if applicable.	**3-5**

Item	Item nr	STROBE recommendations	Extension for Nutritional Epidemiology studies (STROBE-nut)	Reported on page #
Bias	9	Describe any efforts to address potential sources of bias.	**nut-9** Report how bias in dietary or nutritional assessment was addressed, e.g., misreporting, changes in habits as a result of being measured, or data imputation from other sources	**n/a**
Study Size	10	Explain how the study size was arrived at.		**3**
Quantitative variables	11	Explain how quantitative variables were handled in the analyses. If applicable, describe which groupings were chosen and why.	**nut-11** Explain categorization of dietary/nutritional data (e.g., use of N-tiles and handling of nonconsumers) and the choice of reference category, if applicable.	**n/a**
Statistical Methods	12	(a) Describe all statistical methods, including those used to control for confounding<br>(b) Describe any methods used to examine subgroups and interactions.<br>(c) Explain how missing data were addressed.<br>(d) Cohort study—If applicable, explain how loss to follow-up was addressed.<br>Case-control study—If applicable, explain how matching of cases and controls was addressed.<br>Cross-sectional study—If applicable, describe analytical methods taking account of sampling strategy.<br>(e) Describe any sensitivity analyses.	**nut-12.1** Describe any statistical method used to combine dietary or nutritional data, if applicable.<br>**nut-12.2** Describe and justify the method for energy adjustments, intake modeling, and use of weighting factors, if applicable.<br>**nut-12.3** Report any adjustments for measurement error, i.e,. from a validity or calibration study.	**5, 7-8**
**Results**				
Participants	13	(a) Report the numbers of individuals at each stage of the study—e.g., numbers potentially eligible, examined for eligibility, confirmed eligible, included in the study, completing follow-up, and analyzed.<br>(b) Give reasons for non-participation at each stage.<br>(c) Consider use of a flow diagram.	**nut-13** Report the number of individuals excluded based on missing, incomplete or implausible dietary/nutritional data.	**3**

Item	Item nr	STROBE recommendations	Extension for Nutritional Epidemiology studies (STROBE-nut)	Reported on page #
Descriptive data	14	(a) Give characteristics of study participants (e.g., demographic, clinical, social) and information on exposures and potential confounders<br>(b) Indicate the number of participants with missing data for each variable of interest<br>(c) Cohort study—Summarize follow-up time (e.g., average and total amount)	**nut-14** Give the distribution of participant characteristics across the exposure variables if applicable. Specify if food consumption of total population or consumers only were used to obtain results.	**3, 7-8**
Outcome data	15	Cohort study—Report numbers of outcome events or summary measures over time.<br>Case-control study—Report numbers in each exposure category, or summary measures of exposure.<br>Cross-sectional study—Report numbers of outcome events or summary measures.		**7-8**
Main results	16	(a) Give unadjusted estimates and, if applicable, confounder-adjusted estimates and their precision (e.g., 95% confidence interval). Make clear which confounders were adjusted for and why they were included.<br>(b) Report category boundaries when continuous variables were categorized.<br>(c) If relevant, consider translating estimates of relative risk into absolute risk for a meaningful time period.	**nut-16** Specify if nutrient intakes are reported with or without inclusion of dietary supplement intake, if applicable.	**8-9**
Other analyses	17	Report other analyses done—e.g., analyses of subgroups and interactions and sensitivity analyses.	**nut-17** Report any sensitivity analysis (e.g., exclusion of misreporters or outliers) and data imputation, if applicable.	**n/a**
**Discussion**				
Key results	18	Summarize key results with reference to study objectives.		**9, 11, 13**

Item	Item nr	STROBE recommendations	Extension for Nutritional Epidemiology studies (STROBE-nut)	Reported on page #
Limitation	19	Discuss limitations of the study, taking into account sources of potential bias or imprecision. Discuss both direction and magnitude of any potential bias.	**nut-19** Describe the main limitations of the data sources and assessment methods used and implications for the interpretation of the findings.	**20-21**
Interpretation	20	Give a cautious overall interpretation of results considering objectives, limitations, multiplicity of analyses, results from similar studies, and other relevant evidence.	**nut-20** Report the nutritional relevance of the findings, given the complexity of diet or nutrition as an exposure.	**16-20**
Generalizability	21	Discuss the generalizability (external validity) of the study results.		**13**
**Other information**				
Funding	22	Give the source of funding and the role of the funders for the present study and, if applicable, for the original study on which the present article is based.		**15**
Ethics			**nut-22.1** Describe the procedure for consent and study approval from ethics committee(s).	**3, 15**
Supplementary material			**nut-22.2** Provide data collection tools and data as online material or explain how they can be accessed.	**15**

Adapted from Lachat C et al. (2016) [4], “Strengthening the reporting of observational studies in epidemiology-nutritional epidemiology (STROBE-nut): an extension of the STROBE statement,” PLOS Med;13:e1002036. http://dx.doi.org/10.1371/journal.pmed.1002036 pdf or online version.

Table S2. Within- and between-person associations among aspects of sleep (*N* = 590).

		**Sleep outcome**											
**Sleep predictor**	**Analysis**	**1.**	**2.**	**3.**	**4.**	**5.**	**6.**	**7.**	**8.**	**9.**	**10.**	**11.**	**12.**
1. Duration (hrs)	WP (*b*)	---	−.34***	.16***	.63***	−.39***	.03***	---	---	---	---	---	---
	BP (*r*)	---	−.21***	.10*	.34***	−.16***	.05	<.01	−.19***	−.21***	−.12**	.06	−.15**
2. Onset	WP (*b*)	---	---	.77***	.48***	.19***	−.01†	---	---	---	---	---	---
	BP (*r*)	---	---	.95***	.73***	−.02	−.03	.27***	.35***	.27***	.15***	−.29***	.20***
3. Midpoint	WP (*b*)	---	---	---	1.03***	−.09*	.01	---	---	---	---	---	---
	BP (*r*)	---	---	---	.85***	−.07	−.02	.28***	.29***	.21***	.11**	−.28***	.16**
4. Offset	WP (*b*)	---	---	---	---	−.23***	.02***	---	---	---	---	---	---
	BP (*r*)	---	---	---	---	−.11**	−.01	.26***	.29***	.23***	.21***	−.31***	.11*
5. SMeff (%)	WP (*b*)	---	---	---	---	---	.01***	---	---	---	---	---	---
	BP (*r*)	---	---	---	---	---	.02	−.01	.03	.07†	.07†	.16***	.16**
6. Subjective quality[a]	WP (*b*)	---	---	---	---	---	---	---	---	---	---	---	---
	BP (*r*)	---	---	---	---	---	---	−.08*	−.04	−.02	−.04	.12**	−.01
Sleep variability[b]													
7. Duration (*SD*, hrs)	BP (*r*)	---	---	---	---	---	---	---	.41***	.44***	.61***	−.43***	.16**
8. Onset (*SD*, hrs)	BP (*r*)	---	---	---	---	---	---	---	---	.76***	.38***	−.43***	.49***
9. Midpoint (*SD*, hrs)	BP (*r*)	---	---	---	---	---	---	---	---	---	.82***	−.41***	.76***
10. Offset (*SD*, hrs)	BP (*r*)	---	---	---	---	---	---	---	---	---	---	−.37***	.61***
11. SRI[c]	BP (*r*)	---	---	---	---	---	---	---	---	---	---	---	−.24***
12. Social jetlag (hrs)[d]	BP (*r*)	---	---	---	---	---	---	---	---	---	---	---	---

Notes. For within-person effects (WP), values are unstandardized beta coefficients from linear mixed models (*b*). For between-person effects (BP), values are Pearson correlation coefficients (*r*). The between-person effect is represented by each adolescent's mean across all time points. The within-person effect is represented by the deviation from the adolescent's overall mean at each time point. Sleep timing measures (onset, midpoint, and offset) were centered around midnight (0:00). The mean number of valid actigraphy nights per youth was 5.6 ± 1.4 (range: 3-9 nights).

[a]Ranges from 0 (very bad) - 3 (very good).

[b]Between-person only. Higher value means greater variability, except the reverse for the sleep regularity index (SRI).

[c]Calculated based on formula from Phillips et al. [5]; ranges from 0 (low) - 100 (high).

[d]Calculated based on formula from Wittmann et al. [6]. *N* = 372 (adolescent included only if provided at least one weekday and one weekend night of actigraphy; *n* = 372).

b, unstandardized beta; BP, between-person; hrs, hours; *r*, Pearson correlation coefficient; *SD*, standard deviation; SMeff, sleep maintenance efficiency; SRI, sleep regularity index; WP, within-person.

†*p* < .10, **p* < .05, ***p* < .01, ****p* < .001, two-tailed.

REFERENCES

[1] Reichman NE, Teitler J. Fragile Families: Sample and design. Child Youth Serv Rev. 2001;23:303–26.

[2] Wagmiller RLJ. How representative are the Fragile Study families? A comparison of the early childhood longitudinal study-birth cohort and Fragile Families samples. Princet Univ Woodrow Wilson Sch Public Int Aff Cent Res Child Wellbeing, Work Pap. 2010:1–28.

[3] Bendheim-Thoman Center for Research on Child Wellbeing, Columbia Population Research Center. Data user's guide for the Year 15 follow-up wave of the Fragile Families and Child Wellbeing Study 2018:1–63.

[4] Lachat C, Hawwash D, Ocké MC, Berg C, Forsum E, Hörnell A, et al. Strengthening the reporting of observational studies in epidemiology-nutritional epidemiology (STROBE-nut): An extension of the STROBE statement. PLOS Med. 2016;13:e1002036.

[5] Phillips AJK, Clerx WM, O'Brien CS, Sano A, Barger LK, Picard RW, et al. Irregular sleep/wake patterns are associated with poorer academic performance and delayed circadian and sleep/wake timing. Sci Rep. 2017;7:1–13.

[6] Wittmann M, Dinich J, Merrow M, Roenneberg T. Social jetlag: Misalignment of biological and social time. Chronobiol Int. 2006;23:497–509.
